# Excess Relative Risk as an Effect Measure in Case-Control Studies of Rare Diseases

**DOI:** 10.1371/journal.pone.0121141

**Published:** 2015-04-28

**Authors:** Wen-Chung Lee

**Affiliations:** Research Center for Genes, Environment and Human Health, and Institute of Epidemiology and Preventive Medicine, College of Public Health, National Taiwan University, Taipei, Taiwan; University of Michigan Medical School, UNITED STATES

## Abstract

Epidemiologists often use ratio-type indices (rate ratio, risk ratio and odds ratio) to quantify the association between exposure and disease. By comparison, less attention has been paid to effect measures on a difference scale (excess rate or excess risk). The excess relative risk (ERR) used primarily by radiation epidemiologists is of peculiar interest here, in that it involves both difference and ratio operations. The ERR index (but not the difference-type indices) is estimable in case-control studies. Using the theory of sufficient component cause model, the author shows that when there is no mechanistic interaction (no synergism in the sufficient cause sense) between the exposure under study and the stratifying variable, the ERR index (but not the ratio-type indices) in a rare-disease case-control setting should remain constant across strata and can therefore be regarded as a common effect parameter. By exploiting this homogeneity property, the related attributable fraction indices can also be estimated with greater precision. The author demonstrates the methodology (SAS codes provided) using a case-control dataset, and shows that ERR preserves the logical properties of the ratio-type indices. In light of the many desirable properties of the ERR index, the author advocates its use as an effect measure in case-control studies of rare diseases.

## Introduction

Quantifying the association between exposure and disease is a central issue in epidemiology [1]. To this end, epidemiologists often resort to ratio-type indices, such as rate ratio or risk ratio. Under the rare-disease assumption, these indices can be approximated by the odds ratio (also a ratio-type index), which can be conveniently estimated in (cumulative) case-control studies (where controls are selected from noncases at the end of a follow-up period) [1,2]. By comparison, less attention has been paid to effect measures on a difference scale, such as excess rate or excess risk.

In an authoritative textbook, Breslow and Day [3] cited several examples from the literature of cancer epidemiology demonstrating that the ratio-type indices (but not the difference-type) provide a stable measure of association in a wide variety of human populations. In addition to the empirical justifications, they also highlighted the logical properties of the ratio-type indices, stating that they (but not the difference-type indices) are “useful for appraising the extent to which the observed association may be explained by the presence of another agent, or may be specific to a particular disease entity”. For these reasons and their estimability in case-control studies, they are most epidemiologists’ indices of choice when it comes to quantifying exposure-disease associations.

The ‘excess relative risk’ (ERR) used primarily by radiation epidemiologists [4,5] is of peculiar interest here. Compared to the aforementioned indices, ERR is unique in that it involves both difference and ratio operations; it is the excess risk per unit of exposure (difference operation here) divided by the background risk (ratio operation here). ERRs correspond to the beta parameters of a ‘linear relative risk model’ [1,6,7]. For example with two exposures, the model is Risk = exp(*α*) × (1 + *β*_1_ *x*_1_ + *β*_2_ *x*_2_), and the ERRs are *β*_1_, for the first exposure, and *β*_2_, for the second exposure, respectively. The model implies linear dose-response trends and additivity of effects due to different exposures (the addition operations). The model also separates a risk into two parts (the multiplication operation): the background risk, exp(*α*), and the relative risk function, (1 + *β*_1_ *x*_1_ + *β*_2_ *x*_2_). Under the rare-disease assumption, the model becomes a ‘linear odds ratio model’: Odds = exp(*α*) × (1 + *β*_1_ *x*_1_ + *β*_2_ *x*_2_) [1]. Therefore, the beta parameters (that is, the ERRs) are estimable from case-control data. (The ERR index should not be confused with the ‘relative excess risk’ (RER), an index previously proposed by Suissa [8]. RER is a *comparative* effect measure specifically designed to compare the effects of two different exposures (or treatments). In the linear relative risk or linear odds ratio model, RER = *β*_1_/*β*_2_. As this paper focuses on effect measures *proper*, we will not discuss the RER index any further.)

Although the ERR index appears to be a sensible effect measure for a radiation exposure, it has received little attention outside in the field of radiation epidemiology. The purpose of this study is to promote its general use. Using the theory of sufficient component cause model [9–11], I will show that when there is no mechanistic interaction (no synergism in the sufficient cause sense) between the exposure under study and the stratifying variable, the ERR index in a rare-disease case-control study should remain constant across strata and can therefore be regarded as a common effect parameter. (This is true whether the exposure of interest is a radiation agent or not.) As a bonus, I will show that by exploiting this homogeneity property, the related attributable fraction indices can be estimated with greater precision. I will demonstrate the methodology using a case-control dataset. I will also show that ERR preserves the logical properties of the ratio-type indices.

## Methods

### Common Effect Parameters

Consider the relation between an exposure and a disease in a population. The exposure is binary (*E* = 1 indicating a subject is exposed, *E* = 2, otherwise) with an exposure prevalence of *p*) and the disease is assumed to be rare (with a very low disease rate). A stratified analysis is to be performed based on a stratification variable *S* with a total of *L* strata. Let Peril_*s*,*e*_ denote the ‘peril’, and Risk_*s*,*e*_, the disease risk, within a fixed duration of follow-up for subjects with *S* = *s* and *E* = *e* in the study population for *s* = 1,2,…,*L* and *e* = 1,2 [A peril is the reciprocal of a risk complement, that is, Peril_*s*,*e*_ = (1 − Risk_*s*,*e*_)^−1^, see references 12 and 13.] Let Risk_*E* = 1_ (Risk_*E* = 2_) be the marginal disease risk (collapsed over *S*) for the exposed (unexposed) population, with a marginal relative risk (mRR) as $\text{mRR}=\frac{{\text{Risk}}_{E=1}}{{\text{Risk}}_{E=2}}.$

Most epidemiologists are familiar with the concept of *multiplicative* interactions. If there is no multiplicative interaction between the exposure under study and the stratifying variable, we have $\frac{{\text{Risk}}_{s,1}}{{\text{Risk}}_{s,2}}=\text{RR}$, for *s* = 1,2,…,*L*. However, Lee [12, 13] showed that when there is no *mechanistic* interaction between the exposure under study and the stratifying variable, it would be the *peril* ratios (instead of the *risk* ratios) that are constant across strata for a fixed duration of follow-up, that is, $\frac{{\text{Peril}}_{s,1}}{{\text{Peril}}_{s,2}}=\text{PR}$, for *s* = 1,2,…,*L*.

In this paper, we impose the rare-disease assumption. A constant peril ratio therefore implies a constant excess risk (ER), that is, $\text{ER}={\text{Risk}}_{s,1}-{\text{Risk}}_{s,2}\approx \log\frac{{\text{Peril}}_{s,1}}{{\text{Peril}}_{s,2}}=\log\;\text{PR},$ also a constant. The approximation, logPeril = −log(1 − Risk) ≈ Risk, has a bias of less than 0.05% for risk less than 0.001—the setting for studies on cancers, coronary heart diseases, etc. For more common diseases, such as hypertension or type 2 diabetes, the approximation breaks down and the method proposed here would be inapplicable.

The ERR, the focal point of this paper, is

$$\text{ERR}=\frac{{\text{Risk}}_{s,1}-{\text{Risk}}_{s,2}}{{\text{Risk}}_{E=2}}=\frac{\text{ER}}{{\text{Risk}}_{E=2}}.$$

ERR has an additional advantage over ER as an effect measure for the exposure under study; it is not only constant across strata but should be more stable for different durations of follow-up. (By contrast, ER will be roughly doubled if follow-up duration is doubled.) For a homogeneous (unstratified) population, ERR is simply RR minus 1.

For the exposed population, the ‘counterfactual’ disease risk (disease risk when each and every exposed subject, contrary to fact, is unexposed) is its factual disease risk (Risk_*E* = 1_) minus a specific constant, ER, under the constant ERR model. (Note that because the stratification variable *S* may create a confounding effect, the counterfactual disease risk for the exposed population is in general not equal to the factual disease risk for the unexposed population, that is, Risk_*E* = 1_ – ER ≠ Risk_*E* = 2_ in general.) Under the constant ERR model, the population attributable fraction (PAF) is therefore

$$\begin{array}{l} \text{PAF}=\frac{\left(\begin{array}{l} \text{factual disease risk}\\ \text{for the total population} \end{array}\right)-\left(\begin{array}{l} \text{counterfactual disease risk}\\ \text{for the total population}\\ \text{when everyone is unexposed} \end{array}\right)}{\left(\begin{array}{l} \text{factual disease risk}\\ \text{for the total population} \end{array}\right)}\\ =\frac{\left[\begin{array}{l} p\times \left(\begin{array}{l} \text{factual disease risk}\\ \text{for the exposed} \end{array}\right)\\ +(1-p)\times \left(\begin{array}{l} \text{factual disease risk}\\ \text{for the unexposed} \end{array}\right) \end{array}\right]-\left[\begin{array}{l} p\times \left(\begin{array}{l} \text{counterfactual disease risk}\\ \text{for the exposed} \end{array}\right)\\ +(1-p)\times \left(\begin{array}{l} \text{factual disease risk}\\ \text{for the unexposed} \end{array}\right) \end{array}\right]}{\left[\begin{array}{l} p\times \left(\begin{array}{l} \text{factual disease risk}\\ \text{for the exposed} \end{array}\right)\\ +(1-p)\times \left(\begin{array}{l} \text{factual disease risk}\\ \text{for the unexposed} \end{array}\right) \end{array}\right]}\\ =\frac{p\times {\text{Risk}}_{E=1}+(1-p)\times {\text{Risk}}_{E=2}-p\times \left({\text{Risk}}_{E=1}-\text{ER}\right)-(1-p)\times {\text{Risk}}_{E=2}}{p\times {\text{Risk}}_{E=1}+(1-p)\times {\text{Risk}}_{E=2}}\\ =\frac{\text{ERR}}{\text{mRR}+\frac{1-p}{p}}, \end{array}$$

and the attributable fraction among the exposed population (AFE) is

$$\begin{array}{l} \text{AFE}=\frac{\left(\begin{array}{l} \text{factual disease risk}\\ \text{for the exposed} \end{array}\right)-\left(\begin{array}{l} \text{counterfactual disease risk}\\ \text{for the exposed} \end{array}\right)}{\left(\begin{array}{l} \text{factual disease risk}\\ \text{for the exposed} \end{array}\right)}\\ =\frac{{\text{Risk}}_{E=1}-\left({\text{Risk}}_{E=1}-\text{ER}\right)}{{\text{Risk}}_{E=1}}\\ =\frac{\text{ERR}}{\text{mRR}}. \end{array}$$

For a rare disease, PAF and AFE should also remain stable for different durations of follow-up.

### Estimation in Case-Control Studies

The above ERR, PAF and AFE indices (but not the ER index) can be estimated from a case-control study conducted in the population. Assume that a case-control study recruited a total of *n*_1_ cases and *n*_2_ controls. Let CS_*s*,*e*_ (CN_*s*,*e*_) denote the number of cases (controls) with *S* = *s* and *E* = *e* in the case-control sample. Recall that the case-control odds in a case-control study are a constant multiple (the reciprocal of the control sampling fraction of the case-control study, *f*) of the corresponding disease odds (and disease risks for a rare disease) in the population [1]. Therefore, the disease risks for the exposed population, the unexposed population, and subjects with *S* = *s* and *E* = *e* in the study population, can be estimated (if *f* is known) as ${\hat{Risk}}_{E=1}=f\times \frac{{CS}_{+,1}}{{CN}_{+,1}},{\hat{Risk}}_{E=2}=f\times \frac{{CS}_{+,2}}{{CN}_{+,2}}$ and ${\hat{Risk}}_{s,e}=f\times \frac{{CS}_{s,e}}{{CN}_{s,e}},$ respectively, where the plus sign in the subscript indicates a summation over the corresponding index. In general *f* is unknown of course. However, the following three sets of parameters do not depend on *f*:

$$\begin{array}{c} {\hat{\theta }}_{s}=\frac{{\hat{\text{Risk}}}_{s,1}-{\hat{\text{Risk}}}_{s,2}}{{\hat{\text{Risk}}}_{E=2}}\\ ={\hat{\text{OD}}}_{s}\times \frac{{\text{CN}}_{+,2}}{{\text{CS}}_{+,2}}, \end{array}$$

$$\begin{array}{c} {\hat{\psi }}_{s}=\left(\frac{{\hat{\text{Risk}}}_{s,1}-{\hat{\text{Risk}}}_{s,2}}{{\hat{\text{Risk}}}_{E=2}}\right)/\left(\hat{\text{mRR}}+\frac{1-\hat{p}}{\hat{p}}\right)\\ =\left({\hat{\text{OD}}}_{s}\times \frac{{\text{CN}}_{+,2}}{{\text{CS}}_{+,2}}\right)/\left(\frac{{\text{CS}}_{+,1}\times {\text{CN}}_{+,2}}{{\text{CN}}_{+,1}\times {\text{CS}}_{+,2}}+\frac{{\text{CN}}_{+,2}}{{\text{CN}}_{+,1}}\right)\\ ={\hat{\text{OD}}}_{s}\times \frac{{\text{CN}}_{+,1}}{n_{1}}, \end{array}$$

and

$$\begin{array}{c} {\hat{\phi }}_{s}=\left(\frac{{\hat{\text{Risk}}}_{s,1}-{\hat{\text{Risk}}}_{s,2}}{{\hat{\text{Risk}}}_{E=2}}\right)/\hat{\text{mRR}}\\ =\left({\hat{\text{OD}}}_{s}\times \frac{{\text{CN}}_{+,2}}{{\text{CS}}_{+,2}}\right)/\left(\frac{{\text{CS}}_{+,1}\times {\text{CN}}_{+,2}}{{\text{CN}}_{+,1}\times {\text{CS}}_{+,2}}\right)\\ ={\hat{\text{OD}}}_{s}\times \frac{{\text{CN}}_{+,1}}{{\text{CS}}_{+,1}}, \end{array}$$

respectively, where ${\hat{\text{OD}}}_{s}=\frac{{\text{CS}}_{s,1}}{{\text{CN}}_{s,1}}-\frac{{\text{CS}}_{s,2}}{{\text{CN}}_{s,2}}$ is the sample estimate of the odds difference among subjects with *S* = *s* in the case-control data.

### Exploiting the Homogeneity Property

Homogeneity of ERR in the study population implies that the expected values of the odds differences in the case-control data are constant across strata, i.e., $E\left({\hat{OD}}_{s}\right)=OD$, for *s* = 1,2,…,*L*. Therefore, we also have $E\left({\hat{\theta }}_{s}\right)=ERR,E\left({\hat{\psi }}_{s}\right)=PAF,$ and $E\left({\hat{\phi }}_{s}\right)=AFE,$ respectively, for *s* = 1,2,…,*L*. This suggests the following weighted-average estimators for ERR, PAF and AFE:

$$\hat{ERR}=\sum_{s=1}^{L}w_{s}\times {\hat{\theta }}_{s}，$$

$$\hat{PAF}=\sum_{s=1}^{L}u_{s}\times {\hat{\psi }}_{s}，$$

and

$$\hat{AFE}=\sum_{s=1}^{L}v_{s}\times {\hat{\phi }}_{s}，$$

$$\sum_{s=1}^{L}w_{s}=\sum_{s=1}^{L}u_{s}=\sum_{s=1}^{L}v_{s}=1.$$

S1 Exhibit presents the optimal weighting systems (in the sense of minimal variances for the weighted averages) and the variance formulas for these three indices. To set confidence limits, it helps to do the log transformation [*y* = log(1 + *x*) with $\text{Var}(y)=\frac{\text{Var}(x)}{{\left(1+x\right)}^{2}}$] to $\hat{ERR},$ and the complementary log transformation [*z* = −log(1 − *x*) with $\text{Var}(z)=\frac{\text{Var}(x)}{{\left(1-x\right)}^{2}}$] to $\hat{PAF}$ and $\hat{AFE},$ for better approximations. The limits are then transformed back [*x* = exp(*y*) − 1 or *x* = 1 − exp(−*z*)] to the original scale.

The proposed method is based on a constant ERR model (no mechanistic interaction between the exposure under study and the stratifying variable for a rare disease). In practice, this needs to be checked using the data on hand. S2 Exhibit presents a homogeneity test, which is a chi-square test with a degree of freedom of *L* – 1 under the null hypothesis. The test may have low power if the degree of freedom is too large.

If the homogeneity assumption fails, ERR (and ER) will no longer be a meaningful effect measure for the exposure. However, the attributable fraction indices under heterogeneity (${\hat{PAF}}_{het}$ and ${\hat{AFE}}_{het}$) can still be estimated albeit with larger variances, by letting $q_{s}=\frac{{\text{CN}}_{s,1}}{{\text{CN}}_{+,1}}$, the proportion of exposed controls falling in stratum *s*, as the weighting system (S3 Exhibit).

S4 Exhibit presents SAS codes for all the calculations.

## Results

Shapiro et al’s [14] case-control data of myocardial infarction (taken from Table 2–14 in the textbook *Case-Control Studies*: *Design*, *Conduct*, *Analysis* [15]) is reanalyzed here in order to demonstrate the methodologies. The study examined the age-specific relation of myocardial infarction to recent oral contraceptive use (a total of five age strata, see Table 1). The data is consistent with the constant ERR model (p-value = 0.2225; using the chi-squared test in S2 Exhibit).

**Table 1:** Age-specific relation of myocardial infarction to recent oral contraceptive use.

Age	Oral Contraceptive Users			Oral Contraceptive Non-Users			Difference in Case-Control Odds
	Number of Cases	Number of Controls	Case-Control Odds	Number of Cases	Number of Controls	Case-Control Odds	
25–29	4	62	0.0645	2	224	0.0089	0.0556
30–34	9	33	0.2727	12	390	0.0308	0.2420
35–39	4	26	0.1538	33	330	0.1000	0.0538
40–44	6	9	0.6667	65	362	0.1796	0.4871
45–49	6	5	1.2000	93	301	0.3090	0.8910
Total	29	135	0.2148	205	1607	0.1276	0.0872

Table 2 presents the optimal weighting systems under homogeneity (*w*_*s*_ for $\hat{ERR}$, *u*_*s*_ for $\hat{PAF}$ and *v*_*s*_ for $\hat{AFE}$, using the method detailed in S1 Exhibit). From Table 3, we see that the use of oral contraceptive incurs a 57% (95% confidence interval, CI: 16%~112%) increase in myocardial infarction risk. Population-wide, it accounts for 4.8% (95% CI: 1.5% ~ 7.9%) cases, or 54.9% (95% CI: 36.5%~67.9%) exposed cases.

**Table 2:** Weighting systems used for the analysis of the data in Table 1.

Age	*w*_*s*_	*u*_*s*_	*v*_*s*_	*q*_*s*_
25–29	0.7956	0.7529	0.6026	0.4593
30–34	0.0761	0.1032	0.1901	0.2444
35–39	0.1226	0.1282	0.1578	0.1926
40–44	0.0050	0.0119	0.0351	0.0667
45–49	0.0007	0.0038	0.0144	0.0370

**Table 3:** Results for the analysis of the data in Table 1.

Parameter	Estimate	Variance	95% CI^a^
ERR^b^	0.5666	5.8126 E-2	0.1587 ~ 1.1182
PAF^c^	0.0478	2.6436 E-4	0.0154 ~ 0.0792
AFE^d^	0.5488	0.6182 E-2	0.3651 ~ 0.6793

^a^ Confidence Interval.

^b^ Excess Risk Ratio.

^c^ Population Attributable Fraction.

^d^ Attributable Fraction among the Exposed.

The weighting system under heterogeneity (*q*_*s*_) is also presented in Table 2 for comparison. Without exploiting the homogeneity property, it results in much larger variances for the ${\hat{PAF}}_{het}$ (6.6183 E-4 > 2.6436 E-4) and ${\hat{AFE}}_{het}$ (1.3197 E-2 > 0.6182 E-2), as compared to those presented in Table 3 under homogeneity assumption.

## Discussion

Like the commonly used ratio-type indices (rate ratios, risk ratios and odds ratios), ERR also maintains the logical properties of Breslow and Day [3]. Using ERR to quantify association strengths, S5 Exhibit shows that for an observed exposure-disease association to be explained away by an unmeasured factor, the putative factor (if it exists) must be at least as strongly associated with exposure, and also as strongly associated with disease, as that seen between the exposure and disease under study. S6 Exhibit further shows that if the exposure under study is only associated with a specific disease entity, ERR for the exposure and this disease entity will be greater than that for the exposure and the disease as a whole.

S7 Exhibit shows that a constant ERR and a constant risk ratio models cannot be reconciled except for a weak exposure or when disease risks vary little across strata. This raises an interesting proposal that examples of genuine mechanistic interactions may be far more common than we thought—but unfortunately because of the ratio-type indices used, they went unrecognized by previous researchers. Further studies are needed to investigate this postulate. The irreconcilability between the constant ERR and the constant risk ratio models also reveals the inappropriateness of using the risk ratio (or odds ratio) as a measure for exposure effect indiscriminately for all situations, a practice most (if not all) epidemiologists currently follow.

In summary, the ERR index enjoys the logical properties that were previously thought to be exclusive to the ratio-type indices. The ERR index (but not the difference-type indices) is estimable in case-control studies. For rare diseases and in the absence of (sufficient-cause) mechanistic interaction, the ERR index (but not the ratio-type indices) will remain constant across strata and can be regarded as a common effect parameter. Exploiting this homogeneity property, one can also estimate the attributable fraction indices with greater precision. In light of the many desirable properties of the ERR index, the author advocates its use as an effect measure in case-control studies of rare diseases.

## Supporting Information

S1 Exhibit Optimal weighting systems and variance formulas for excess relative risk (ERR), population attributable fraction (PAF) and attributable fraction among the exposed population (AFE). (PDF)

S2 Exhibit A homogeneity test. (PDF)

S3 Exhibit Estimation of population attributable fraction (PAF) and attributable fraction among the exposed population (AFE) under heterogeneity. (PDF)

S4 Exhibit SAS codes. (PDF)

S5 Exhibit A proof that for an observed exposure-disease association to be explained away by an unmeasured factor, the putative factor (if it exists) must be at least as strongly associated with exposure, and also as strongly associated with disease, as that seen between the exposure and disease under study. (PDF)

S6 Exhibit A proof that if the exposure under study is only associated with a specific disease entity, excess relative risk (ERR) for the exposure and this disease entity will be greater than that for the exposure and the disease as a whole. (PDF)

S7 Exhibit A proof that a constant excess relative risk (ERR) and a constant risk ratio (RR) models cannot be reconciled except for a weak exposure or when disease risks vary little across strata. (PDF)
