# Supplementary material for: Excess Relative Risk as an Effect Measure in Case-Control Studies of Rare Diseases
Source: PLoS One. 2015 Apr 28;10(4):e0121141. doi: 10.1371/journal.pone.0121141 (PMC4412639; doi:10.1371/journal.pone.0121141)
Supplement: S1 Exhibit — (PDF) [file pone.0121141.s001.pdf]

Supporting Information of

Excess Relative Risk as an Effect Measure in Case-Control Studies of Rare Diseases

Author: Wen-Chung Lee

Author's affiliation: Research Center for Genes, Environment and Human Health,
and Institute of Epidemiology and Preventive Medicine,
College of Public Health, National Taiwan University, Taipei, Taiwan.

Correspondence & reprint requests: Prof. Wen-Chung Lee,
Rm. 536, No. 17, Xuzhou Rd., Taipei 100, Taiwan.
(FAX: 886-2-23511955)
(e-mail: wenchung@ntu.edu.tw)

S1 Exhibit. Optimal weighting systems and variance formulas for excess relative risk (ERR), population attributable fraction (PAF) and attributable fraction among the exposed population (AFE).

Let $\widehat{\mathbf{A}}$ and $\widehat{\mathbf{B}}$ denote the $L \times 2$ matrices, the (s, e)-th elements of which being the sample proportions of subjects with $S = s$ and $E = e$ in the case ($\hat{a}_{s,e}$) and the control ($\hat{b}_{s,e}$) groups, respectively. Assuming that the cells counts are distributed according to two independent multinomial distributions (one for the cases and the other, the controls), the asymptotic variance-covariance matrices (of dimension, $2L \times 2L$) are

$$\text{Var } \widehat{\mathbf{A}} = \text{Var}\left(\text{vec } \widehat{\mathbf{A}}\right) = \frac{1}{n_1} \times \left[\text{Diag}\left(\text{vec } \widehat{\mathbf{A}}\right) - \left(\text{vec } \widehat{\mathbf{A}}\right)\left(\text{vec } \widehat{\mathbf{A}}\right)^{\text{t}}\right],$$

$$\text{Var } \widehat{\mathbf{B}} = \text{Var}\left(\text{vec } \widehat{\mathbf{B}}\right) = \frac{1}{n_2} \times \left[\text{Diag}\left(\text{vec } \widehat{\mathbf{B}}\right) - \left(\text{vec } \widehat{\mathbf{B}}\right)\left(\text{vec } \widehat{\mathbf{B}}\right)^{\text{t}}\right],$$

and

$$\text{Cov}\left(\widehat{\mathbf{A}}, \widehat{\mathbf{B}}\right) = \mathbf{0},$$

respectively.

Let $\hat{\boldsymbol{\theta}}$, $\hat{\boldsymbol{\psi}}$, and $\hat{\boldsymbol{\varphi}}$ be $L \times 1$ vectors, with the sth element of which being

$$\hat{\theta}_s = \left(\frac{a_{s,1}}{b_{s,1}} - \frac{a_{s,2}}{b_{s,2}}\right) \times \frac{b_{+,2}}{a_{+,2}},$$

$$\hat{\psi}_s = \left(\frac{a_{s,1}}{b_{s,1}} - \frac{a_{s,2}}{b_{s,2}}\right) \times b_{+,1},$$

and

$$\hat{\phi}_s = \left(\frac{a_{s,1}}{b_{s,1}} - \frac{a_{s,2}}{b_{s,2}}\right) \times \frac{b_{+,1}}{a_{+,1}},$$

respectively. Their variance-covariance matrices can be obtained using the multivariate delta

method:

$$\mathrm{Var}\ \hat{\boldsymbol{\theta}} = \left(\frac{\partial \hat{\boldsymbol{\theta}}}{\partial \hat{\mathbf{A}}}\right)^{\mathrm{t}} \left(\mathrm{Var}\ \hat{\mathbf{A}}\right)\left(\frac{\partial \hat{\boldsymbol{\theta}}}{\partial \hat{\mathbf{A}}}\right) + \left(\frac{\partial \hat{\boldsymbol{\theta}}}{\partial \hat{\mathbf{B}}}\right)^{\mathrm{t}} \left(\mathrm{Var}\ \hat{\mathbf{B}}\right)\left(\frac{\partial \hat{\boldsymbol{\theta}}}{\partial \hat{\mathbf{B}}}\right),$$

$$\mathrm{Var}\ \hat{\boldsymbol{\psi}} = \left(\frac{\partial \hat{\boldsymbol{\psi}}}{\partial \hat{\mathbf{A}}}\right)^{\mathrm{t}} \left(\mathrm{Var}\ \hat{\mathbf{A}}\right)\left(\frac{\partial \hat{\boldsymbol{\psi}}}{\partial \hat{\mathbf{A}}}\right) + \left(\frac{\partial \hat{\boldsymbol{\psi}}}{\partial \hat{\mathbf{B}}}\right)^{\mathrm{t}} \left(\mathrm{Var}\ \hat{\mathbf{B}}\right)\left(\frac{\partial \hat{\boldsymbol{\psi}}}{\partial \hat{\mathbf{B}}}\right),$$

and

$$\mathrm{Var}\ \hat{\boldsymbol{\varphi}} = \left(\frac{\partial \hat{\boldsymbol{\varphi}}}{\partial \hat{\mathbf{A}}}\right)^{\mathrm{t}} \left(\mathrm{Var}\ \hat{\mathbf{A}}\right)\left(\frac{\partial \hat{\boldsymbol{\varphi}}}{\partial \hat{\mathbf{A}}}\right) + \left(\frac{\partial \hat{\boldsymbol{\varphi}}}{\partial \hat{\mathbf{B}}}\right)^{\mathrm{t}} \left(\mathrm{Var}\ \hat{\mathbf{B}}\right)\left(\frac{\partial \hat{\boldsymbol{\varphi}}}{\partial \hat{\mathbf{B}}}\right),$$

respectively. Here the differentiation of a vector with respect to a matrix follows the convention: $\frac{\partial \mathbf{y}}{\partial \mathbf{X}} = \frac{\partial \mathbf{y}^{\mathrm{t}}}{\partial(\mathrm{vec}\ \mathbf{X})}$ and has a dimension of $2L \times L$.

Let $I_{\mathrm{statement}}$ be an indicator function with a value of 1, if the statement is true, and 0, if otherwise. For $i \in \{1,...,L\}$, $j \in \{1,2\}$, and $k \in \{1,...,L\}$, the ($i + L \times j - L$, k)-th elements of the derivative matrices are

$$\left(\frac{I_{(i=k,j=1)}}{b_{k,1}} - \frac{I_{(i=k,j=2)}}{b_{k,2}} - \frac{I_{(j=2)} \times \hat{\theta}_k}{b_{+,2}}\right) \times \frac{b_{+,2}}{a_{+,2}} \quad \text{for} \quad \frac{\partial \hat{\boldsymbol{\theta}}}{\partial \hat{\mathbf{A}}},$$

$$\frac{I_{(j=2)} \times \hat{\theta}_k}{b_{+,2}} - \left(\frac{I_{(i=k,j=1)} \times a_{k,1}}{b_{k,1}^2} - \frac{I_{(i=k,j=2)} \times a_{k,2}}{b_{k,2}^2}\right) \times \frac{b_{+,2}}{a_{+,2}} \quad \text{for} \quad \frac{\partial \hat{\boldsymbol{\theta}}}{\partial \hat{\mathbf{B}}},$$

$$\left(\frac{I_{(i=k,j=1)}}{b_{k,1}} - \frac{I_{(i=k,j=2)}}{b_{k,2}}\right) \times b_{+,1} \quad \text{for} \quad \frac{\partial \hat{\boldsymbol{\psi}}}{\partial \hat{\mathbf{A}}},$$

$$\frac{I_{(j=1)} \times \hat{\psi}_k}{b_{+,1}} - \left(\frac{I_{(i=k,j=1)} \times a_{k,1}}{b_{k,1}^2} - \frac{I_{(i=k,j=2)} \times a_{k,2}}{b_{k,2}^2}\right) \times b_{+,1} \quad \text{for} \quad \frac{\partial \hat{\boldsymbol{\psi}}}{\partial \hat{\mathbf{B}}},$$

$$\left(\frac{I_{(i=k,j=1)}}{b_{k,1}} - \frac{I_{(i=k,j=2)}}{b_{k,2}} - \frac{I_{(j=1)} \times \hat{\phi}_k}{b_{+,1}}\right) \times \frac{b_{+,1}}{a_{+,1}} \quad \text{for} \quad \frac{\partial \hat{\boldsymbol{\varphi}}}{\partial \hat{\mathbf{A}}},$$

and

$$\frac{I_{(j=1)} \times \hat{\phi}_k}{b_{+,1}} - \left(\frac{I_{(i=k,j=1)} \times a_{k,1}}{b_{k,1}^2} - \frac{I_{(i=k,j=2)} \times a_{k,2}}{b_{k,2}^2} \right) \times \frac{b_{+,1}}{a_{+,1}} \quad \text{for} \quad \frac{\partial \hat{\boldsymbol{\varphi}}}{\partial \hat{\mathbf{B}}},$$

respectively.

The estimates for ERR, PAF and AFE are

$$\widehat{\text{ERR}} = \mathbf{w}^{\text{t}} \hat{\boldsymbol{\theta}},$$

$$\widehat{\text{PAF}} = \mathbf{u}^{\text{t}} \hat{\boldsymbol{\psi}},$$

and

$$\widehat{\text{AFE}} = \mathbf{v}^{\text{t}} \hat{\boldsymbol{\varphi}},$$

respectively, with the weighting vectors subject to $\mathbf{w}^{\text{t}}\mathbf{1} = \mathbf{u}^{\text{t}}\mathbf{1} = \mathbf{v}^{\text{t}}\mathbf{1} = 1$, where $\mathbf{1}$ is the summing vector (a '1' for each and every element). Using the extended Cauchy-Schwarz inequality (see Johnson RA, Wichern DW. Applied Multivariate Statistical Analysis. 3rd ed. New Jersey: Prentice-Hall International; 1992), the variances are

$$\text{Var}\left(\widehat{\text{ERR}}\right) = \mathbf{w}^{\text{t}} \left(\text{Var}\ \hat{\boldsymbol{\theta}}\right) \mathbf{w} \geq \frac{1}{\mathbf{1}^{\text{t}} \left(\text{Var}\ \hat{\boldsymbol{\theta}}\right)^{-1} \mathbf{1}},$$

$$\text{Var}\left(\widehat{\text{PAF}}\right) = \mathbf{u}^{\text{t}} \left(\text{Var}\ \hat{\boldsymbol{\psi}}\right) \mathbf{u} \geq \frac{1}{\mathbf{1}^{\text{t}} \left(\text{Var}\ \hat{\boldsymbol{\psi}}\right)^{-1} \mathbf{1}},$$

and

$$\text{Var}\left(\widehat{\text{AFE}}\right) = \mathbf{v}^{\text{t}} \left(\text{Var}\ \hat{\boldsymbol{\varphi}}\right) \mathbf{v} \geq \frac{1}{\mathbf{1}^{\text{t}} \left(\text{Var}\ \hat{\boldsymbol{\varphi}}\right)^{-1} \mathbf{1}},$$

with equalities if and only if $\mathbf{w} = \frac{\left(\text{Var}\ \hat{\boldsymbol{\theta}}\right)^{-1} \mathbf{1}}{\mathbf{1}^{\text{t}} \left(\text{Var}\ \hat{\boldsymbol{\theta}}\right)^{-1} \mathbf{1}}$, $\mathbf{u} = \frac{\left(\text{Var}\ \hat{\boldsymbol{\psi}}\right)^{-1} \mathbf{1}}{\mathbf{1}^{\text{t}} \left(\text{Var}\ \hat{\boldsymbol{\psi}}\right)^{-1} \mathbf{1}}$, and

$\mathbf{v} = \frac{\left(\text{Var}\ \hat{\boldsymbol{\varphi}}\right)^{-1} \mathbf{1}}{\mathbf{1}^{\text{t}} \left(\text{Var}\ \hat{\boldsymbol{\varphi}}\right)^{-1} \mathbf{1}}$, respectively.
