# Supplementary material for: Excess Relative Risk as an Effect Measure in Case-Control Studies of Rare Diseases
Source: PLoS One. 2015 Apr 28;10(4):e0121141. doi: 10.1371/journal.pone.0121141 (PMC4412639; doi:10.1371/journal.pone.0121141)
Supplement: S2 Exhibit — (PDF) [file pone.0121141.s002.pdf]

Supporting Information of

# Excess Relative Risk as an Effect Measure in Case-Control Studies of Rare Diseases

Author: Wen-Chung Lee

Author's affiliation: Research Center for Genes, Environment and Human Health,

and Institute of Epidemiology and Preventive Medicine,

College of Public Health, National Taiwan University, Taipei, Taiwan.

Correspondence & reprint requests: Prof. Wen-Chung Lee,

Rm. 536, No. 17, Xuzhou Rd., Taipei 100, Taiwan.

(FAX: 886-2-23511955)

(e-mail: wenchung@ntu.edu.tw)

S2 Exhibit. A homogeneity test.

For a rare disease, testing the additivity of the case-control odds in the case-control data is tantamount to testing a constant ERR in the study population. Let $\hat{\boldsymbol{\delta}}$ be a $(L-1)\times 1$ column vector with the $s$ th element being

$$\begin{aligned}\hat{\delta}_s &= \widehat{\mathrm{OD}}_s - \widehat{\mathrm{OD}}_L \\ &= \frac{a_{s,1}}{b_{s,1}} - \frac{a_{s,2}}{b_{s,2}} - \frac{a_{L,1}}{b_{L,1}} + \frac{a_{L,2}}{b_{L,2}}.\end{aligned}$$

Here $\hat{\delta}_s$ are the estimates of the differences in the odds differences, using the odds difference of the $L$ th stratum as the reference. (Any stratum can be taken to be the reference, with the same testing result.)

The variance-covariance matrix [of a dimension of $(L-1)\times(L-1)$] of $\hat{\boldsymbol{\delta}}$ can be obtained using the multivariate delta method:

$$\mathrm{Var}\ \hat{\boldsymbol{\delta}} = \left(\frac{\partial\hat{\boldsymbol{\delta}}}{\partial\widehat{\mathbf{A}}}\right)^{\mathrm{t}} \left(\mathrm{Var}\ \widehat{\mathbf{A}}\right)\left(\frac{\partial\hat{\boldsymbol{\delta}}}{\partial\widehat{\mathbf{A}}}\right) + \left(\frac{\partial\hat{\boldsymbol{\delta}}}{\partial\widehat{\mathbf{B}}}\right)^{\mathrm{t}} \left(\mathrm{Var}\ \widehat{\mathbf{B}}\right)\left(\frac{\partial\hat{\boldsymbol{\delta}}}{\partial\widehat{\mathbf{B}}}\right).$$

For $i\in\{1,...,L\}$, $j\in\{1,2\}$, and $k\in\{1,...,L-1\}$, the ($i+L\times j-L$, $k$)-th elements of the derivative matrices are

$$\frac{I_{(i=k,j=1)}}{b_{k,1}} - \frac{I_{(i=k,j=2)}}{b_{k,2}} - \frac{I_{(i=L,j=1)}}{b_{L,1}} + \frac{I_{(i=L,j=2)}}{b_{L,2}} \quad \text{for } \frac{\partial\hat{\boldsymbol{\delta}}}{\partial\widehat{\mathbf{A}}},$$

and

$$-\frac{I_{(i=k,j=1)}\times a_{k,1}}{b_{k,1}^2} + \frac{I_{(i=k,j=2)}\times a_{k,2}}{b_{k,2}^2} + \frac{I_{(i=L,j=1)}\times a_{L,1}}{b_{L,1}^2} - \frac{I_{(i=L,j=2)}\times a_{L,2}}{b_{L,2}^2} \quad \text{for } \frac{\partial\hat{\boldsymbol{\delta}}}{\partial\widehat{\mathbf{B}}},$$

respectively. Next, the following statistic is calculated:

$$T = \hat{\boldsymbol{\delta}}^{\mathbf{t}}\left(\mathrm{Var}\ \hat{\boldsymbol{\delta}}\right)^{-1}\hat{\boldsymbol{\delta}},$$

which is distributed as a chi-square distribution with $L-1$ degree of freedom under the null hypothesis of a constant ERR in the study population (no mechanistic interaction between the exposure under study and the stratifying variable for a rare disease).
