# Supplementary material for: Excess Relative Risk as an Effect Measure in Case-Control Studies of Rare Diseases
Source: PLoS One. 2015 Apr 28;10(4):e0121141. doi: 10.1371/journal.pone.0121141 (PMC4412639; doi:10.1371/journal.pone.0121141)
Supplement: S3 Exhibit — (PDF) [file pone.0121141.s003.pdf]

Supporting Information of

Excess Relative Risk as an Effect Measure in Case-Control Studies of Rare Diseases

Author: Wen-Chung Lee

Author's affiliation: Research Center for Genes, Environment and Human Health,

and Institute of Epidemiology and Preventive Medicine,

College of Public Health, National Taiwan University, Taipei, Taiwan.

Correspondence & reprint requests: Prof. Wen-Chung Lee,

Rm. 536, No. 17, Xuzhou Rd., Taipei 100, Taiwan.

(FAX: 886-2-23511955)

(e-mail: wenchung@ntu.edu.tw)

S3 Exhibit. Estimation of population attributable fraction (PAF) and attributable fraction among the exposed population (AFE) under heterogeneity.

Under heterogeneity,

$$
\begin{aligned}
\widehat{\mathrm{AFE}}_{\mathrm{het}} &= \frac{\widehat{\mathrm{Risk}}_{E=1} - \sum_{s=1}^{L} \widehat{\Pr}\left(S=s \mid E=1\right) \times \widehat{\mathrm{Risk}}_{s,2}}{\widehat{\mathrm{Risk}}_{E=1}} \\
&= \frac{f \times \dfrac{\mathrm{CS}_{+,1}}{\mathrm{CN}_{+,1}} - \sum_{s=1}^{L} \dfrac{\mathrm{CN}_{s,1}}{\mathrm{CN}_{+,1}} \times f \times \dfrac{\mathrm{CS}_{s,2}}{\mathrm{CN}_{s,2}}}{f \times \dfrac{\mathrm{CS}_{+,1}}{\mathrm{CN}_{+,1}}} \\
&= \left(\mathrm{CS}_{+,1} - \sum_{s=1}^{L} \frac{\mathrm{CS}_{s,2} \times \mathrm{CN}_{s,1}}{\mathrm{CN}_{s,2}} \right) \Big/ \mathrm{CS}_{+,1} \\
&= \sum_{s=1}^{L} \frac{\mathrm{CN}_{s,1}}{\mathrm{CN}_{+,1}} \times \left(\frac{\mathrm{CS}_{s,1}}{\mathrm{CN}_{s,1}} - \frac{\mathrm{CS}_{s,2}}{\mathrm{CN}_{s,2}} \right) \times \frac{\mathrm{CN}_{+,1}}{\mathrm{CS}_{+,1}} \\
&= \sum_{s=1}^{L} \frac{\mathrm{CN}_{s,1}}{\mathrm{CN}_{+,1}} \times \hat{\phi}_s .
\end{aligned}
$$

Using Miettinen's case-based formula (see Miettinen OS. Proportion of disease caused or prevented by a given exposure, trait or intervention. Am J Epidemiol. 1974;99:325–332),

$$
\begin{aligned}
\widehat{\mathrm{PAF}}_{\mathrm{het}} &= (\text{exposure prevalence among cases}) \times \widehat{\mathrm{AFE}}_{\mathrm{het}} \\
&= \frac{\mathrm{CS}_{+,1}}{n_1} \times \sum_{s=1}^{L} \frac{\mathrm{CN}_{s,1}}{\mathrm{CN}_{+,1}} \times \left(\frac{\mathrm{CS}_{s,1}}{\mathrm{CN}_{s,1}} - \frac{\mathrm{CS}_{s,2}}{\mathrm{CN}_{s,2}} \right) \times \frac{\mathrm{CN}_{+,1}}{\mathrm{CS}_{+,1}} \\
&= \sum_{s=1}^{L} \frac{\mathrm{CN}_{s,1}}{\mathrm{CN}_{+,1}} \times \left(\frac{\mathrm{CS}_{s,1}}{\mathrm{CN}_{s,1}} - \frac{\mathrm{CS}_{s,2}}{\mathrm{CN}_{s,2}} \right) \times \frac{\mathrm{CN}_{+,1}}{n_1} \\
&= \sum_{s=1}^{L} \frac{\mathrm{CN}_{s,1}}{\mathrm{CN}_{+,1}} \times \hat{\psi}_s .
\end{aligned}
$$
