# Supplementary material for: Excess Relative Risk as an Effect Measure in Case-Control Studies of Rare Diseases
Source: PLoS One. 2015 Apr 28;10(4):e0121141. doi: 10.1371/journal.pone.0121141 (PMC4412639; doi:10.1371/journal.pone.0121141)
Supplement: S4 Exhibit — (PDF) [file pone.0121141.s004.pdf]

Supporting Information of

Excess Relative Risk as an Effect Measure in Case-Control Studies of Rare Diseases

Author: Wen-Chung Lee

Author's affiliation: Research Center for Genes, Environment and Human Health,
and Institute of Epidemiology and Preventive Medicine,
College of Public Health, National Taiwan University, Taipei, Taiwan.

Correspondence & reprint requests: Prof. Wen-Chung Lee,
Rm. 536, No. 17, Xuzhou Rd., Taipei 100, Taiwan.
(FAX: 886-2-23511955)
(e-mail: wenchung@ntu.edu.tw)

S4 Exhibit. SAS codes.

```
proc iml;
/********************* Input your data here ****************************(****/
CS_exp={4   9   4    6    6};          /**** Number of Exposed Cases      *****/
CS_unexp={2  12  33  65  93};         /**** Number of Unexposed Cases    *****/
CN_exp={62  33  26   9   5};          /**** Number of Exposed Controls   *****/
CN_unexp={224  390  330  362  301};   /**** Number of Unexposed Controls *****/
/****************************************************************************/
L=ncol(CS_exp); L1=L - 1; twoL=2#L;
n1=CS_exp[+] + CS_unexp[+];
n2=CN_exp[+] + CN_unexp[+];
A=( CS_exp` || CS_unexp` ) / n1;
B=( CN_exp` || CN_unexp` ) / n2;
vecA=A[,1]//A[,2];
vecB=B[,1]//B[,2];
VarA=( Diag(vecA) - vecA * vecA` ) / n1;
VarB=( Diag(vecB) - vecB * vecB` ) / n2;
a_p1=A[+,1]; a_p2=A[+,2];
b_p1=B[+,1]; b_p2=B[+,2];
odds_1=A[,1] / B[,1];
odds_2=A[,2] / B[,2];
diff=odds_1 - odds_2;
cscn_1=odds_1#n1/n2;
cscn_2=odds_2#n1/n2;
OD=diff#n1/n2;
theta=diff#(b_p2 / a_p2);
psi=diff#b_p1;
phi=diff#(b_p1 / a_p1);
delta=diff-j(L,1, diff[L]);
Theta_A=j(twoL, L, 0); Theta_B=j(twoL, L, 0);
Psi_A=j(twoL, L, 0); Psi_B=j(twoL, L, 0);
Phi_A=j(twoL, L, 0); Phi_B=j(twoL, L, 0);
Delta_A=j(twoL, L, 0); Delta_B=j(twoL, L, 0);
one=j(L, 1, 1);
do i=1 to L;
do j=1 to 2;
```

```
do k=1 to L;
     index= i + L#j - L;
     Theta_A[index, k]=
          ( (i=k)#(j=1)/B[k,1] - (i=k)#(j=2)/B[k,2] - (j=2)#theta[k]/b_p2 ) # (b_p2/a_p2);
     Theta_B[index, k]=
          (j=2)#theta[k]/b_p2
            -( (i=k)#(j=1)#A[k,1]/B[k,1]##2 - (i=k)#(j=2)#A[k,2]/B[k,2]##2 ) # (b_p2/a_p2);
     Psi_A[index, k]=
          ( (i=k)#(j=1)/B[k,1] - (i=k)#(j=2)/B[k,2] ) # b_p1;
     Psi_B[index, k]=
          (j=1)#psi[k]/b_p1
            -( (i=k)#(j=1)#A[k,1]/B[k,1]##2 - (i=k)#(j=2)#A[k,2]/B[k,2]##2 ) # b_p1;
     Phi_A[index, k]=
          ( (i=k)#(j=1)/B[k,1] - (i=k)#(j=2)/B[k,2] - (j=1)#phi[k]/b_p1 ) # (b_p1/a_p1);
      Phi_B[index, k]=
          (j=1)#phi[k]/b_p1
            -( (i=k)#(j=1)#A[k,1]/B[k,1]##2 - (i=k)#(j=2)#A[k,2]/B[k,2]##2 ) # (b_p1/a_p1);
     Delta_A[index, k]=
          (i=k)#(j=1)/B[k,1] - (i=k)#(j=2)/B[k,2] +  (i=L)#(j=1)/B[L,1] - (i=L)#(j=2)/B[L,2];
      Delta_B[index, k]=
           - (i=k)#(j=1)#A[k,1]/B[k,1]##2 + (i=k)#(j=2)#A[k,2]/B[k,2]##2
         + (i=L)#(j=1)#A[L,1]/B[L,1]##2 - (i=L)#(j=2)#A[L,2]/B[L,2]##2;
end;end;end;
delta=delta[1:L1];
Delta_A=Delta_A[, 1:L1];
Delta_B=Delta_B[, 1:L1];
Var_Theta=Theta_A` * VarA * Theta_A + Theta_B` * VarB * Theta_B;
Var_Psi=Psi_A` * VarA * Psi_A + Psi_B` * VarB * Psi_B;
Var_Phi=Phi_A` * VarA * Phi_A + Phi_B` * VarB * Phi_B;
Var_Delta=Delta_A` * VarA * Delta_A + Delta_B` * VarB * Delta_B;
w= ( inv(Var_Theta) * one ) / ( one` * inv(Var_Theta) * one );
u= ( inv(Var_Psi) * one ) / ( one` * inv(Var_Psi) * one );
v= ( inv(Var_Phi) * one ) / ( one` * inv(Var_Phi) * one );
q=B[,1]/b_p1;
reset nocenter;
print 'Weighting Systems';
print '     ' w [format=12.4] u [format=12.4] v [format=12.4] q [format=12.4];
ERR=w` * theta;
PAF=u` * psi;
```

```
AFE=v` * phi;
PAF_het=q` * psi;
AFE_het=q` * phi;
Var_ERR=1 / ( one` * inv(Var_Theta) * one );
Var_PAF=1 / ( one` * inv(Var_Psi) * one );
Var_AFE=1 / ( one` * inv(Var_Phi) * one );
Var_PAF_het= q` * Var_Psi * q;
Var_AFE_het= q` * Var_Phi * q;
print 'Variances';
print '     ' Var_ERR [format=16.8] Var_PAF [format=16.8] Var_AFE [format=16.8]
        Var_PAF_het [format=16.8] Var_AFE_het [format=16.8];
crit=probit(0.975);
t_Var_ERR=Var_ERR / (1 + ERR)##2;
temp1=log(1+ERR) - crit #sqrt(t_Var_ERR);
temp2=log(1+ERR) + crit #sqrt(t_Var_ERR);
ERR_low= exp(temp1) - 1;
ERR_upp= exp(temp2) - 1;
t_Var_PAF=Var_PAF / (1 - PAF)##2;
temp1= - log(1-PAF) - crit #sqrt(t_Var_PAF);
temp2= - log(1-PAF) + crit #sqrt(t_Var_PAF);
PAF_low=1 - exp( - temp1);
PAF_upp=1 - exp( - temp2);
t_Var_AFE=Var_AFE / (1 - AFE)##2;
temp1= - log(1-AFE) - crit #sqrt(t_Var_AFE);
temp2= - log(1-AFE) + crit #sqrt(t_Var_AFE);
AFE_low=1 - exp( - temp1);
AFE_upp=1 - exp( - temp2);
t_Var_PAF_het=Var_PAF_het / (1 - PAF_het)##2;
temp1= - log(1-PAF_het) - crit #sqrt(t_Var_PAF_het);
temp2= - log(1-PAF_het) + crit #sqrt(t_Var_PAF_het);
PAF_het_low=1 - exp( - temp1);
PAF_het_upp=1 - exp( - temp2);
t_Var_AFE_het=Var_AFE_het / (1 - AFE_het)##2;
temp1= - log(1-AFE_het) - crit #sqrt(t_Var_AFE_het);
temp2= - log(1-AFE_het) + crit #sqrt(t_Var_AFE_het);
AFE_het_low=1 - exp( - temp1);
AFE_het_upp=1 - exp( - temp2);
print 'Estimates and 95% Confidence Intervals';
print '     ' ERR [format=12.4] ERR_low [format=12.4] ERR_upp [format=12.4];
```

```
print '      ' PAF [format=12.4] PAF_low [format=12.4] PAF_upp [format=12.4];

print '      ' AFE [format=12.4] AFE_low [format=12.4] AFE_upp [format=12.4];

print '      ' PAF_het [format=12.4] PAF_het_low [format=12.4] PAF_het_upp [format=12.4];

print '      ' AFE_het [format=12.4] AFE_het_low [format=12.4] AFE_het_upp [format=12.4];

T=delta` * inv(Var_Delta) * delta;

p=1-probchi(T, L1);

reset noname;

print 'Testing for Heterogeneity';

print '    Test Statistic = ' T [format=12.4];

print '    Degree of Freedom = ' L1 [format=5.0];

print '    P-Value = ' p [format=12.4];
```
