# Supplementary material for: Excess Relative Risk as an Effect Measure in Case-Control Studies of Rare Diseases
Source: PLoS One. 2015 Apr 28;10(4):e0121141. doi: 10.1371/journal.pone.0121141 (PMC4412639; doi:10.1371/journal.pone.0121141)
Supplement: S5 Exhibit — (PDF) [file pone.0121141.s005.pdf]

Supporting Information of

Excess Relative Risk as an Effect Measure in Case-Control Studies of Rare Diseases

Author: Wen-Chung Lee

Author's affiliation: Research Center for Genes, Environment and Human Health,

and Institute of Epidemiology and Preventive Medicine,

College of Public Health, National Taiwan University, Taipei, Taiwan.

Correspondence & reprint requests: Prof. Wen-Chung Lee,

Rm. 536, No. 17, Xuzhou Rd., Taipei 100, Taiwan.

(FAX: 886-2-23511955)

(e-mail: wenchung@ntu.edu.tw)

S5 Exhibit. A proof that for an observed exposure-disease association to be explained away by an unmeasured factor, the putative factor (if it exists) must be at least as strongly associated with exposure, and also as strongly associated with disease, as that seen between the exposure and disease under study.

Assumed that the exposure under study (*E*) has no effect whatsoever on the occurrence of the disease (*D*), except for the possible confounding effect from a binary confounder (*U*) which is unmeasured in the study. The excess risk quantifying the relation between *U* and *E* is $\mathrm{ER}_{UE} = \Pr(U=1 \mid E=1) - \Pr(U=1 \mid E=2)$, and the excess risk quantifying the relation between *U* and *D* is $\mathrm{ER}_{UD} = \Pr(D=1 \mid U=1) - \Pr(D=1 \mid U=2)$, respectively. The observed disease risks are then $p_U \times (r + \mathrm{ER}_{UD}) + (1 - p_U) \times r$ in the unexposed population, and $(p_U + \mathrm{ER}_{UE}) \times (r + \mathrm{ER}_{UD}) + (1 - p_U - \mathrm{ER}_{UE}) \times r$ in the exposed population, respectively, where $p_U = \Pr(U=1 \mid E=2)$ is the proportion of people with $U=1$ in the unexposed population and $r = \Pr(D=1 \mid U=2)$ is the disease risk for people with $U=2$. The apparent excess risk for the relation between *E* and *D* is

$$\begin{aligned}\mathrm{ER}_{ED} &= \Pr(D=1 \mid E=1) - \Pr(D=1 \mid E=2)\\ &= (p_U + \mathrm{ER}_{UE}) \times (r + \mathrm{ER}_{UD}) + (1 - p_U - \mathrm{ER}_{UE}) \times r\\ &\quad - p_U \times (r + \mathrm{ER}_{UD}) - (1 - p_U) \times r\\ &= \mathrm{ER}_{UE} \times \mathrm{ER}_{UD},\end{aligned}$$

and the apparent excess risk ratio,

$$\mathrm{ERR}_{ED} = \frac{\mathrm{ER}_{UE} \times \mathrm{ER}_{UD}}{\Pr(D=1 \mid E=2)}.$$

Assuming $\mathrm{ER}_{UE} > 0$ and $\mathrm{ER}_{UD} > 0$ (positive associations between *U* and *E* and between *U* and *D*), the following two inequalities are derived:

$$\begin{aligned}
\mathrm{ERR}_{ED} &= \frac{\mathrm{ER}_{UE} \times \mathrm{ER}_{UD}}{\Pr(D=1 \mid E=2)} \\
&< \frac{\mathrm{ER}_{UD}}{\Pr(D=1 \mid E=2)} \\
&= \frac{\mathrm{ER}_{UD}}{\Pr(U=1 \mid E=2) \times \Pr(D=1 \mid U=1) + \Pr(U=2 \mid E=2) \times \Pr(D=1 \mid U=2)} \\
&< \frac{\mathrm{ER}_{UD}}{\Pr(U=1 \mid E=2) \times \Pr(D=1 \mid U=2) + \Pr(U=2 \mid E=2) \times \Pr(D=1 \mid U=2)} \\
&= \frac{\mathrm{ER}_{UD}}{\Pr(D=1 \mid U=2)} \\
&= \mathrm{ERR}_{UD}
\end{aligned}$$

and

$$\begin{aligned}
\mathrm{ERR}_{ED} &= \frac{\mathrm{ER}_{UE} \times \mathrm{ER}_{UD}}{\Pr(D=1 \mid E=2)} \\
&= \frac{\mathrm{ER}_{UE} \times \left[\Pr(D=1 \mid U=1) - \Pr(D=1 \mid U=2)\right]}{\Pr(U=1 \mid E=2) \times \Pr(D=1 \mid U=1) + \Pr(U=2 \mid E=2) \times \Pr(D=1 \mid U=2)} \\
&< \frac{\mathrm{ER}_{UE} \times \Pr(D=1 \mid U=1)}{\Pr(U=1 \mid E=2) \times \Pr(D=1 \mid U=1)} \\
&= \mathrm{ERR}_{UE}.
\end{aligned}$$
