# Supplementary material for: Excess Relative Risk as an Effect Measure in Case-Control Studies of Rare Diseases
Source: PLoS One. 2015 Apr 28;10(4):e0121141. doi: 10.1371/journal.pone.0121141 (PMC4412639; doi:10.1371/journal.pone.0121141)
Supplement: S6 Exhibit — (PDF) [file pone.0121141.s006.pdf]

Supporting Information of

Excess Relative Risk as an Effect Measure in Case-Control Studies of Rare Diseases

Author: Wen-Chung Lee

Author's affiliation: Research Center for Genes, Environment and Human Health,
and Institute of Epidemiology and Preventive Medicine,
College of Public Health, National Taiwan University, Taipei, Taiwan.

Correspondence & reprint requests: Prof. Wen-Chung Lee,
Rm. 536, No. 17, Xuzhou Rd., Taipei 100, Taiwan.
(FAX: 886-2-23511955)
(e-mail: wenchung@ntu.edu.tw)

S6 Exhibit. A proof that if the exposure under study is only associated with a specific disease entity, excess relative risk (ERR) for the exposure and this disease entity will be greater than that for the exposure and the disease as a whole.

Assumed that the disease under study is composed of two disease entities (D_{I} and D_{II}) and that the exposure under study (*E*) has no effect whatsoever on the occurrence of D_{II}, that is, $\Pr(D_{\mathrm{II}}=1 \mid E=1)=\Pr(D_{\mathrm{II}}=1 \mid E=2)$. We see that the excess risk ratio quantifying the relation between *E* and *D* (the D_{I} and D_{II} combined) is less than that between *E* and D_{I}:

$$
\begin{aligned}
\mathrm{ERR}_{\mathrm{I+II}} &= \frac{\Pr(D=1 \mid E=1)-\Pr(D=1 \mid E=2)}{\Pr(D=1 \mid E=2)} \\
&= \frac{\left[\Pr(D_{\mathrm{I}}=1 \mid E=1)+\Pr(D_{\mathrm{II}}=1 \mid E=1)\right]-\left[\Pr(D_{\mathrm{I}}=1 \mid E=2)+\Pr(D_{\mathrm{II}}=1 \mid E=2)\right]}{\Pr(D_{\mathrm{I}}=1 \mid E=2)+\Pr(D_{\mathrm{II}}=1 \mid E=2)} \\
&= \frac{\Pr(D_{\mathrm{I}}=1 \mid E=1)-\Pr(D_{\mathrm{I}}=1 \mid E=2)}{\Pr(D_{\mathrm{I}}=1 \mid E=2)+\Pr(D_{\mathrm{II}}=1 \mid E=2)} \\
&< \frac{\Pr(D_{\mathrm{I}}=1 \mid E=1)-\Pr(D_{\mathrm{I}}=1 \mid E=2)}{\Pr(D_{\mathrm{I}}=1 \mid E=2)} \\
&= \mathrm{ERR}_{\mathrm{I}}.
\end{aligned}
$$
