# Supplementary material for: Excess Relative Risk as an Effect Measure in Case-Control Studies of Rare Diseases
Source: PLoS One. 2015 Apr 28;10(4):e0121141. doi: 10.1371/journal.pone.0121141 (PMC4412639; doi:10.1371/journal.pone.0121141)
Supplement: S7 Exhibit — (PDF) [file pone.0121141.s007.pdf]

Supporting Information of

Excess Relative Risk as an Effect Measure in Case-Control Studies of Rare Diseases

Author: Wen-Chung Lee

Author's affiliation: Research Center for Genes, Environment and Human Health,

and Institute of Epidemiology and Preventive Medicine,

College of Public Health, National Taiwan University, Taipei, Taiwan.

Correspondence & reprint requests: Prof. Wen-Chung Lee,

Rm. 536, No. 17, Xuzhou Rd., Taipei 100, Taiwan.

(FAX: 886-2-23511955)

(e-mail: wenchung@ntu.edu.tw)

S7 Exhibit. A proof that a constant excess relative risk (ERR) and a constant risk ratio (RR) models cannot be reconciled except for a weak exposure or when disease risks vary little across strata.

Under a constant ERR model, we have $\text{RR}_s = \dfrac{\text{Risk}_{s,2} + \text{ER}}{\text{Risk}_{s,2}} = 1 + \dfrac{\text{Risk}_{E=2} \times \text{ERR}}{\text{Risk}_{s,2}}$.

Therefore,

$$\begin{aligned}\text{Var}\left(\text{RR}_s\right) &= \text{Var}\left(\frac{\text{Risk}_{E=2} \times \text{ERR}}{\text{Risk}_{s,2}}\right)\\ &= \frac{\text{Risk}_{E=2}^2 \times \text{ERR}^2}{\text{Risk}_{E=2}^4} \times \text{Var}\left(\text{Risk}_{s,2}\right)\\ &= \text{ERR}^2 \times \text{CV}^2\left(\text{Risk}_{s,2}\right),\end{aligned}$$

where $\text{CV}\left(\text{Risk}_{s,2}\right) = \dfrac{\sqrt{\text{Var}\left(\text{Risk}_{s,2}\right)}}{\text{Risk}_{E=2}}$ is the coefficient of variation of the disease risks of the unexposed population across the strata. Under a constant RR model, we have

$\text{ERR}_s = \dfrac{\text{RR} \times \text{Risk}_{s,2} - \text{Risk}_{s,2}}{\text{Risk}_{E=2}} = \dfrac{\left(\text{RR} - 1\right)}{\text{Risk}_{E=2}} \times \text{Risk}_{s,2}$. Therefore,

$$\begin{aligned}\text{Var}\left(\text{ERR}_s\right) &= \text{Var}\left[\frac{\left(\text{RR} - 1\right)}{\text{Risk}_{E=2}} \times \text{Risk}_{s,2}\right]\\ &= \frac{\left(\text{RR} - 1\right)^2}{\text{Risk}_{E=2}^2} \times \text{Var}\left(\text{Risk}_{s,2}\right)\\ &= \left(\text{RR} - 1\right)^2 \times \text{CV}^2\left(\text{Risk}_{s,2}\right).\end{aligned}$$
